# Challenges and Promise for Glioblastoma Treatment through Extracellular Vesicle Inquiry †

**DOI:** 10.3390/cells13040336

**Published:** 2024-02-13

**Authors:** Giovanna L. Liguori

**Affiliations:** Institute of Genetics and Biophysics (IGB) “Adriano Buzzati-Traverso”, National Research Council (CNR) of Italy, 80131 Naples, Italy; giovanna.liguori@igb.cnr.it

**Keywords:** glioblastoma, extracellular vesicles, tumor therapy, drug delivery

## Abstract

Glioblastoma (GB) is a rare but extremely aggressive brain tumor that significantly impacts patient outcomes, affecting both duration and quality of life. The protocol established by Stupp and colleagues in 2005, based on radiotherapy and chemotherapy with Temozolomide, following maximum safe surgical resection remains the gold standard for GB treatment; however, it is evident nowadays that the extreme intratumoral and intertumoral heterogeneity, as well as the invasiveness and tendency to recur, of GB are not compatible with a routine and unfortunately ineffective treatment. This review article summarizes the main challenges in the search for new valuable therapies for GB and focuses on the impact that extracellular vesicle (EV) research and exploitation may have in the field. EVs are natural particles delimited by a lipidic bilayer and filled with functional cellular content that are released and uptaken by cells as key means of cell communication. Furthermore, EVs are stable in body fluids and well tolerated by the immune system, and are able to cross physiological, interspecies, and interkingdom barriers and to target specific cells, releasing inherent or externally loaded functionally active molecules. Therefore, EVs have the potential to be ideal allies in the fight against GB and to improve the prognosis for GB patients. The present work describes the main preclinical results obtained so far on the use of EVs for GB treatment, focusing on both the EV sources and molecular cargo used in the various functional studies, primarily in vivo. Finally, a SWOT analysis is performed, highlighting the main advantages and pitfalls of developing EV-based GB therapeutic strategies. The analysis also suggests the main directions to explore to realize the possibility of exploiting EVs for the treatment of GB.

## 1. Introduction

Gliomas are the most frequent intracranial tumors in adults. The first reports of gliomas date back to the early 19th century thanks to the British scientists Berns and Abernethy; however, the first comprehensive histomorphological description was made by the German pathologist Rudolf Virchow in 1865. Studying gliomas, Virchow first introduced the concept of “neuroglia”, from which gliomas derive, as a connective tissue of the brain and the spinal cord that is formed by star-shaped units, interconnected by fine fibers, in which the nervous elements are located [1,2]. The term glioblastoma (GB) was first used in 1927 by the neuropathologist Percival Bailey and the neurosurgeon Harvey Cushing, who developed the first systematic classification and histological description of gliomas, providing the basis for the modern classification of gliomas [3]. Since then, glioma classification has been updated several times by the World Health Organization (WHO), introducing different nomenclature and diagnostic criteria. In the fourth edition of the WHO Classification of CNS Tumors (WHO CNS4), the term “multiforme” was abolished [4]. Furthermore, in the WHO CNS4, and even more so in the fifth and latest 2021 edition (WHO CNS5), besides the traditionally used histological and immunohistochemical features, molecular (genetic and expression data) parameters were also incorporated, establishing a different approach to both CNS tumor nomenclature and grading, and highlighting the significance of integrated diagnoses [5,6,7]. 

According to the WHO Classification of CNS Tumors, GB is the highest and most severe prognostic-grade, namely “grade IV”, glioma and the most aggressive and lethal among all primary brain tumors, with a median overall survival without treatment of at most 4 months, otherwise it is 14–17 months after diagnosis, and it has a 5-year survival rate of 5–6% [4,5,8,9]. Although considered a rare tumor, with an incidence rate of approximately 3 per 100,000 people, GB accounted for 14.5% of all CNS tumors and for 48.6% of the malignant ones based on the data collected in the Central Brain Tumor Registry of the United States (CBTRUS) from 2014 to 2018 [8]. The tumor has a slightly higher incidence in men than in women and a higher incidence in Caucasian than in African or Asian populations. The annual age-adjusted incidence of GB increases with age from 0.15 per 100,000 in children to 15.03 per 100,000 in patients over 75, with a median age at diagnosis of 65 years. In the pediatric age range, however, although rare, GB constitutes one of the groups of neoplasms with the worst prognosis [8,10]. Unfortunately, recent studies indicate an increase in the incidence of both GB and brain tumors in general, as highlighted by the Global Cancer Statistics of 2020 [11]. The etiology of GB is unknown, with the only identifiable risk factor being exposure to ionizing radiation. Other possible contributing factors include aging, non-ionizing radiation, and air pollution [12,13]. 

GB primarily develops in the cerebral hemispheres. Depending on the affected brain area and increased intracranial pressure, patients may exhibit a variety of rapidly developing and life-altering symptoms. Neurological symptoms, such as severe headache, loss of vision, language alteration, and persistent weakness, as well as psychological and psychiatric symptoms, including unpredictable personality changes, can severely compromise the quality of life and even the autonomy of patients, with a strong impact on the patient‘s family and friends [14,15,16,17]. Diagnosis typically occurs only at the onset of symptoms and is primarily based on magnetic resonance imaging (MRI), which has technical limitations in terms of specificity (difficulties in excluding other diagnosis) and sensitivity (spatial resolution of 2–3 mm) [18]. The most widely adopted therapy for GB since 2005 involves the surgical removal of the tumor mass, followed by radiotherapy and chemotherapy, according to the protocol identified by Stupp and coworkers [19] (Stupp et al., 2005). The surgical removal of GB is a complex procedure due to the large number of cells that make up the tumor and their high infiltration power in surrounding healthy tissues. This is a major cause of the high recurrence rate, and the success of the procedure is strongly dependent on the location and accessibility of the tumor mass. Extensive surgical therapy, where possible, is crucial to improve patient prognosis and reduce the risk of recurrence, although this may increase the risk of postoperative neurological deficits [20]. After surgical resection, and if this is not possible, patients receive radiotherapy in combination with chemotherapy, of which Temozolomide (TMZ), a DNA alkylating agent, is the chemotherapy of choice [19]. 

Recently, clinical trials have tested alternative therapies, such as extended adjuvant TMZ treatment, the monoclonal anti-VEGFA antibody Bevacizumab, the receptor tyrosine kinases inhibitor Regorafenib, magnetic tumor-treating fields, and immunotherapies, alone or in combination with established treatments [21,22,23,24]. Moreover, in order to identify the most effective interventions, several meta-analyses have compared the efficacy of different treatments across different randomized clinical trials. These studies have highlighted some promising results, such as the ability of Bevacizumab in combination with TMZ to slightly increase progression-free survival (but not overall patient survival), making it a potential salvage regimen for recurrent GB [25,26,27,28].

Novel approaches based on photodynamic and sonodynamic therapies, also in combination, have shown promising results in preclinical studies of GB, both in vitro and in vivo [29,30,31,32,33]. Photodynamic therapy (PDT) induces cell death in the target tissue through the production and accumulation of reactive oxygen species by a combination of light energy, oxygen, and photosensitizing cytotoxic agents [29,34,35]. Recent studies have shown that phytocompounds, such as berberine and curcumin, can be used as efficient photosensitizers in PDT against highly malignant gliomas [29,36,37]. Sonodynamic therapy (SDT) is a noninvasive procedure that uses focused ultrasounds to activate sonosensitizers (i.e., 5-Aminolevulinic Acid or 5-ALA) to block tumor growth, although the mechanism is still unclear [32]. These approaches provide the valuable opportunity for spatially and temporally specific GB treatments with limited systemic toxicity. Several clinical trials are ongoing or have recently ended to evaluate the feasibility and effectiveness of PDT (NCT01966809 terminated; NCT05363826, NCT04391062, NCT04469699, and NCT03897491 recruiting; and NCT05736406 not yet recruiting) and SDT (NCT04845919, NCT06039709, NCT05370508, and NCT05362409, all recruiting) for GB treatments. The results of these studies will be fundamental for the integration of these therapies into clinical practice.

So far, no breakthrough therapies leading to the extensive and durable survival of patients have been discovered, and survival rates for GB patients have not shown a notable improvement in population-based studies [10]. Therefore, there is a significant need for effective and minimally invasive diagnostic and therapeutic tools to detect early GB onset, and, even more, to block GB progression and avoid its recurrence after surgery. 

Improving our understanding of GB onset and progression at the molecular, cellular, and systemic levels is critical in identifying new key therapeutic targets and implementing effective treatment strategies. In this scenario, the discovery of natural and functional nanoparticles surrounded by a lipid membrane, called extracellular vesicles (EVs), which are released and uptaken by cells in both physiological and pathological conditions, including tumorigenesis, results in the strong potential for inspiring new, more effective, and less invasive diagnostic and therapeutic approaches for GB patient treatment. In this review, we will focus on the significance of EVs in the development of new promising therapeutic strategies against GB. 

## 2. Main Issues in GB Therapy

**Figure 1:**
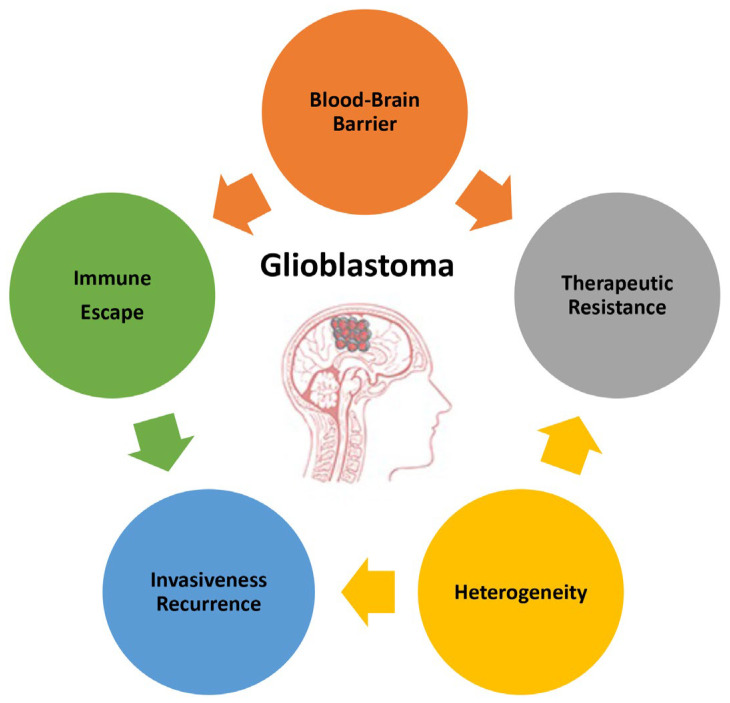
Main features of glioblastoma responsible for its high malignancy and poor prognosis.

The poor prognosis of GB and the objective difficulties in developing successful therapies are due to the complex and unique features characterizing this type of tumor. This paragraph summarizes the main attributes of GB (Figure 1) to highlight the direction that basic investigations, research and development, and technology transfers should take.

### 2.1. Blood–Brain Barrier

The blood–brain barrier (BBB) is a highly selective and specialized barrier that maintains the homeostasis of the CNS, protecting and isolating the brain from potentially harmful agents transported in the blood, such as viruses and other pathogens. The BBB has a complex structure formed by multiple layers, including brain microvascular non-fenestrated endothelial cells sealed by junctional complexes, and is surrounded by the end feet of glial cells on the outer face. The physiological barrier is the result of a series of physical, transport, and metabolic processes within endothelial cells that are highly coordinated by interactions with different vascular, immune, and neural cell types [38,39,40]. Therefore, the BBB is not only a physical barrier, but it also actively uses endothelial efflux transporters to pump small lipophilic molecules that can diffuse through the plasma membrane back in the bloodstream. Even though the BBB plays an evolutionarily important role in preserving brain health and function, in the case of brain pathologies, such as GB, it can hinder the delivery of therapeutic molecules to the brain, limiting their clinical success [39,40,41]. Moreover, as the barrier works in both directions, the flow of molecules from the brain to the bloodstream is also restricted, reducing the diagnostic potential of liquid biopsies based on blood samples for brain diseases [18].

Interestingly, the integrity of the barrier is compromised in many brain pathologies, including GB; however, this alone is not enough to enhance drug accumulation and efficacy. This is in part due to the highly heterogeneous barrier disruption and the persistence of an intact barrier in areas surrounding the tumor [40,42,43,44]. Additionally, in the case of GB, the impaired BBB, also known as the blood–tumor barrier (BTB), can function synergistically with the intact BBB to create a tumor-supportive adaptive environment, paradoxically allowing for the transport of pro-tumoral molecules while limiting the entry of antitumoral ones [45,46]. 

### 2.2. Heterogeneity

GB is a highly dynamic and rapidly evolving tumor characterized by strong molecular and cellular heterogeneity within an individual tumor at a given time, as well as at different times during tumor development and even more so in different patients [47,48]. The high inter- and intratumoral heterogeneity of GB can affect tumor diagnosis and characterization, as it is challenging to achieve a comprehensive description of the whole molecular signature in such a heterogeneous tumor through surgical biopsies. To have a better chance of accurate diagnosis, multiple biopsies should be performed and repeated during the progression of GB, a practice that is neither feasible nor sustainable for the patient [18]. Moreover, GBs’ heterogeneity makes it extremely difficult to target all tumor cells inside the same patient and to cure different patients with the same drug [49]. With regard to GB, we can distinguish between molecular and a cellular heterogeneity. 

#### 2.2.1. Molecular

In 2016, the WHO CNS4 classified gliomas into low-grade gliomas (LGGs) and GB, with GB further subdivided into primary and secondary tumors [4]. Primary and secondary GBs are histologically indistinguishable, but develop from different genetic precursors and therefore show different genetic alterations. Primary, or de novo, GB is characterized by the absence of a mutation in the *isocitrate dehydrogenase 1* (*IDH1*) gene, accounts for 90% of all GBs, affects older patients, and has a worse prognosis. The most frequent genetic alterations include the loss of heterozygosity (LOH) on chromosome 10q, mutations or deletions in the *Phosphatase and Tensin Homolog* (*PTEN*) gene, the mutation or amplification of the *Epidermal Growth Factor Receptor* (*EGFR*) gene, and the deletion of the *Cyclin-Dependent Kinase Inhibitor 2A/B (CDKN2A/B)* gene. Secondary GB, arising from LGGs or astrocytomas, are rarer, usually diagnosed at a younger age, and have a more favorable outcome. The most common genetic alterations include *IDH1* gene mutations, an LOH at 22q and 19q chromosomes, and the methylation of the *O6 Methylguanine-DNA Methyltransferase* (*MGMT*) promoter [50].

GBs’ molecular signature was identified and listed in The Cancer Genome Atlas (TCGA) project, which reported a large-scale multi-dimensional analysis at the genomic, epigenomic, and transcriptomic level [51,52]. These studies highlighted frequent alterations in core oncogenic pathways involving the tumor protein p53, the receptor tyrosine kinase/Ras/phosphoinositide 3-kinase, and retinoblastoma. Furthermore, a bulk gene expression profile analysis identified three main GB molecular subtypes, namely proneural (TCGA-PN), classical (TCGA-CL), and mesenchymal (TCGA-MES), harboring mutations in the *Platelet-derived growth factor receptor A* (*PDGFRA*) and *IDH1* genes (PN), *EGFR* gene (MES), and *Neurofibromatosis 1* (*NF1*) gene (CL), respectively, with PN having a better prognosis than the MES subtype [48,53]. However, over time, the switching of a GB tumor from one subtype to another has been described, e.g., from PN to MES, and is one of the main causes of resistance to treatments [54,55]. Recently, single-cell RNA sequencing pointed out the high level of intratumoral complexity by showing the presence of transcriptionally distinct subclones, each with specific features, in the same tumor sample [56]. 

#### 2.2.2. Cellular

A second layer of heterogeneity is due to the developmental state of GB cells in the tumor. GBs contain subsets of GB stem cells (GSCs) that resemble and could be derived from neural stem cells (NSCs) [57,58,59,60]. GSCs are considered to represent the driving force of GBs, as they are able to maintain stemness and proliferation, as well as to remain quiescent and escape both chemotherapy and radiotherapy. These abilities can be transferred to other tumor cells, conditioning the tumor microenvironment to support GB growth, progression, and recurrence [61]. GSCs could be isolated thanks to the presence of CD133, CD44, and L1 cell adhesion molecules (L1CAMs) on the cell surface, but it is unknown whether different GSC markers isolate different cellular states or subtypes and whether tumors generated with different subpopulations of GSCs give rise to GBs with a comparable or distinct cellular composition. Several studies have shown that GSCs in recurrent GB differ from those that initiated and maintained in the primary tumor, displaying different surface markers (CD15, BMI1, and SOX2, instead of CD133) and being more aggressive. Finally, GB single-cell sequencing analyses suggested that tumor cell hierarchy arising from GSCs might account for the intratumoral cellular and molecular heterogeneity [46,62,63].

### 2.3. Invasiveness and Recurrence

GB cells have been shown to invade normal brain tissues, penetrating the brain parenchyma and the perivascular space by degrading the extracellular matrix through the release of proteolytic factors and by forming membrane-derived extensions known as invadopodia, which are involved in cell migration [64,65]. The patterns, directionality, and mechanisms responsible for this invasive behavior involve the extracellular matrix (ECM) and cytoskeletal remodeling, cell-to-cell and cell-to-ECM adhesion mechanisms, and the overall features of epithelial–mesenchymal transition (EMT). GB cells show a preference toward specific brain regions (the subventricular zone) compared to others, such as the hippocampus and cerebellum. Due to their aggressive migratory behavior, GB cells can escape complete surgical resection, resulting in recurrence within a few centimeters of the original location. Almost all GB tumors return after surgery, radiotherapy, and chemotherapy, and show a lower response rate to conventional therapies, leaving very few options of treatment. Currently, management protocols for recurrent GB patients are still lacking, and most patients are not eligible for surgical re-resection [66]. Multiple studies have identified the tumor-initiating and clonogenic potential of GSCs, as well as their role in therapeutic resistance, making them the main contributors to GB recurrence. After debulking, the unremoved GSCs migrate within the resection cavity and initiate and recapitulate the entire tumor [67,68,69]. 

### 2.4. Therapeutic Resistance

Therapeutic resistance is one of the main causes of poor prognosis for patients with GB. This feature increases over time, weakening current therapeutic protocols for recurrent GB. In most cases, surgery is no longer possible, and chemo- and radiotherapy fail to counteract tumor progression [46]. Many of the features of GB described here have also been linked to radio- and/or chemoresistance. First of all, the presence of the BBB limits the access of chemotherapeutics to the brain [40]. Moreover, therapeutic resistance tends to increase over time due to the molecular changes that occur during GB progression, including the switching from the PN to the MES subtype, and the accumulation of O6-methylguanine-DNA methyltransferase (MGMT). MGMT is in charge of DNA repair, and increased levels of MGMT are associated with increased resistance to TMZ chemotherapy [70]. GSCs play a key role in therapeutic resistance, showing low sensitivity to both chemo- and radiotherapies, which further decreases following repeated chemoradiation treatments [71,72]. Therapeutic resistance is mediated by the increase in MGMT, as well as anti-apoptotic proteins drug-efflux transporters, which reduce drug sensitivity, and the activation of both DNA damage checkpoints and DNA repair mechanism induced by radiation [73,74].

### 2.5. Immune Escape

The immunosurveillance of the CNS is naturally designed to preserve neuronal function and minimize non-specific immune responses. Additionally, GBs have been described as an immunologically “cold” tumor that employs multiple immunosuppressive mechanisms [75]. GB cells present few new tumoral immunogenic antigens on the surface, likely due to a low mutational burden, the post-transcriptional downregulation of antigen expression, and, last but not least, the post-translational mechanisms that interfere with antigen exposure on the cell membrane. This is the case for the family of ligands of the Natural Killer group 2D (NKG2D) receptor, which act as stress-induced ligands expressed by damaged or transformed cells to be recognized and cleared by immune cells. The ligands bind and activate the NKG2D receptor on NK cells, triggering cytotoxicity, and T cell differentiation and expansion [76]. However, GB cells have various mechanisms to limit the expression and/or the exposure on the membrane of NGK2 ligands and to escape immune surveillance. These mechanisms include translational regulation by miRNAs, intracellular retention, proteolytic degradation, as well as shedding from the membrane through proteolytic cleavage or EV encapsulation and release [76]. Moreover, GB cells contribute to creating an immunosuppressive microenvironment by actively secreting soluble factors, interleukins, prostaglandins, and EVs that target microglia, monocytes, and macrophages, thereby inducing a tumor-supportive M2 phenotype; as well as T lymphocytes, sequestering them in the bone marrow and impairing their antitumoral activity. The complex GB microenvironment, conditioned by the GB itself, facilitates the ability of the tumor to evade the immune response, to progress, and also to recur [46,77,78,79,80]. Immunotherapy against GB is still challenging due to its characteristics. To date, clinical trials involving immune checkpoint inhibitors, vaccine-based therapy, and viral therapy have not yielded the desirable positive outcomes observed in other tumors. Ongoing studies are investigating different combinatorial approaches to overcome GB features [80].

## 3. Investigating EVs for GB Treatment: Strategies, Advantages, and Future Perspectives

GB cells communicate with each other and with the tumor microenvironment. EVs play key roles in this process, as they transfer functional molecules (DNA, mRNAs, microRNAs or miRNAs, long non-coding RNAs or lncRNAs, proteins, lipids, and metabolites) and their related properties between cells [81]. EVs are released by cells, mainly through two mechanisms that involve budding from the cell membrane (microvesicles and ectosomes), or the exocytosis of intraluminal vesicles formed inside the multivesicular bodies present in the cytoplasm (exosomes) [82,83,84]. The first mechanism produces both small and large EVs, while the second one specifically gives rise to smaller vesicles due to physical cellular constraints [85]. EVs can travel through the bloodstream and other biological fluids, transporting the messengers contained within over a considerable distance from the site of origin. They can target specific cells through cell membrane interactions and can be internalized in the target cells, where they release their functional content [85,86,87]. 

**Figure 2:**
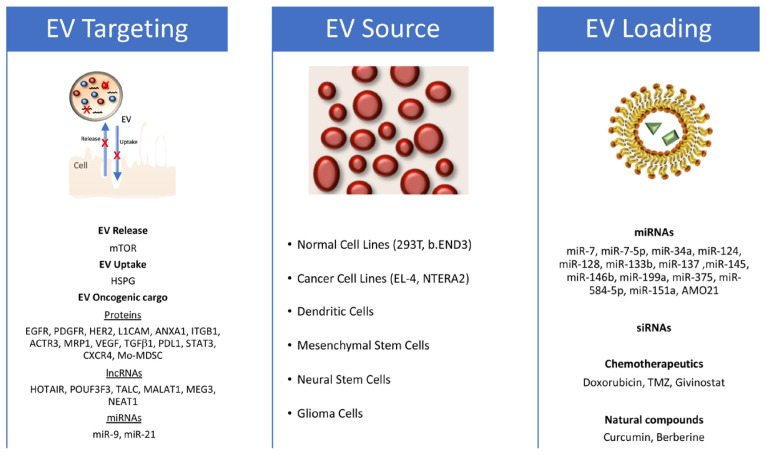
EV exploitation strategy for the treatment of glioblastoma. Figure schematizes the identified targets that block EV release and uptake or impair specific oncogenic glioblastoma (GB) features on the left; the EV sources used in different approaches against GB in the middle; and the therapeutic molecules used in functional assays in glioblastoma models on the right (both in vitro and in vivo).

The release of EVs by GB cells, particularly GSCs, can promote the oncogenic transformation of normal brain cells and increase the malignancy of other GB cells by inducing proliferation, motility, and chemoresistance. Additionally, GB-derived EVs (GDEVs) can target and condition non-tumoral cells in the GB microenvironment, such as macrophages, immune cells, astrocytes, and endothelial cells, inducing angiogenesis and immune escape, and creating a supportive microenvironment for GB growth and progression. In turn, cells in the conditioned tumor microenvironment release EVs that are uptaken by GB cells, increasing their proliferation, motility, invasiveness, and resistance to therapy. Several review articles have explored the role played by EVs in the cellular cross-talk within GB microenvironments [65,81,88,89]. This review, instead, aims to focus on how EV biology can be exploited to overcome current limitations in GB therapy and develop innovative and breakthrough therapeutic approaches (Figure 2). 

### 3.1. Targeting EV Biology and Cargo

EVs are very much implicated in the establishment of a supportive microenvironment for GB, as well as tumors in general, including their progression and the induction of key malignant features, such as proliferation, invasiveness, chemoresistance, and immune escape. Therefore, therapeutic strategies aimed at blocking EV release, uptake, and circulation may counteract GB progression and improve patient outcomes, regardless of the high tumor heterogeneity. 

Several strategies have been identified that are based on targeting EVs on route (i.e., hemodialysis) or inhibiting EV release and uptake, including the use of natural substances (i.e., the antifungal agent ketoconazole or the alkaloid berberine extracted from herbal plants), as well as based on repurposed drugs such as heparin and reserpine, which are already used as anticoagulant and anti-hypertensive drugs, respectively [64,90,91,92]. Alone, berberine, an efficient sensitizer used in PDT, can also suppress cancer cell proliferation by reducing the synthesis of fatty acids and inhibiting the formation and secretion of extracellular vesicles in cancer cells [91]. 

In glioma cells heparan sulfate proteoglycans (HSPGs) have been identified as key modulators of EV uptake [93]. A decrease in HSPGs on the cell membrane of GBs and the use of free heparin, which competes with cell-membrane HSPGs for the binding to EVs, strongly reduces EV uptake [94]; however, the heparin-mediated blocking of EV binding to target cells and the subsequent internalization is not specific to cancer cells [95,96]. Identifying selective strategies to address tumor-derived EV biology, without affecting normal EVs, might be fundamental for developing clinically suitable, effective, and safe interventions, avoiding undesired off-target effects. In GB, increased levels of the mammalian Target of Rapamycin (mTOR) and consequent pathway activation are required for promoting EV release through the downregulation of the autophagy pathway, as well as for maintaining GSC tumor features (self-renewal, proliferation, migration, and invasiveness) [67]. This mechanism is potentially relevant for the development of novel approaches that selectively target GDEV production. 

In parallel, defining specific functionally relevant GB-associated EV cargo that regulates the complex crosstalk within GB environment may allow for specific and targeted therapeutic approaches. Several GDEV-associated proteins have been identified as key players in the induction of the proliferation (EGFRvIII, PDGFR, and Human Epidermal Growth Factor Receptor 2 (HER2)), invasiveness (L1CAM, Annexin A1 or ANXA1, Integrin Beta 1 (ITB1), and Actin-related protein 3 (ACTR3)), and chemoresistance (multidrug resistance protein 1 (MRP1)) of GB cells. GDEVs are also enriched in pro-angiogenic factors, such as vascular endothelial growth factor (VEGF), transforming growth factor beta 1 (TGFβ1), C-X-C chemokine receptor type 4 (CXCR4), and plasminogen activators and proteases, which induce endothelial cells proliferation and tube formation as well as in immune modulators, such as Programmed Cell Death Ligand 1 (PDL1) and monocytic myeloid-derived suppressor cells (Mo-MDSC), which inhibit inflammatory and immune responses. In the last decade, gene/RNA therapy has gained significant attention in the GB therapeutic field. Several lncRNAs with oncogenic activity have been found inside GDEVs [97]. Among these, the lncRNAs POUF3F3 and TALC can remodel the GB microenvironment. POUF3F3 acts on endothelial cells, inducing angiogenesis and GB progression, while TALC acts on microglia, inducing M2 polarization with the consequent secretion of the complement components C5/C5a, leading to the induction of TMZ chemoresistance in GB cells [98,99]. Other GDEV-associated lncRNAs, such as MALAT1, MEG3, NEAT1, and HOTAIR, play a key role in promoting EMT and possibly chemoresistance when uptaken by GB cells both in vitro and in vivo [97]. There is accumulating evidence that miRNAs also play a significant role in GB pathogenesis and have a high potential for use in targeted therapeutic approaches [100]. Among the others, miR-9, upregulated in GSCs, has been found in EVs isolated from both GB cell cultures and patients, and is strongly implied in the phenotypic traits of malignant GBs including cell proliferation and migration [101]. miR-9 is also involved in the expression of the drug efflux protein P-glycoprotein, which contributes to an increase in TMZ drug resistance in GB cells. Anti-miR-9 molecules encapsulated in EVs derived from Mesenchymal Stem Cells (MSCs) were shown to be successfully delivered to GB cells in vitro, causing a reduction in the levels of P-glycoprotein and enhancing TMZ sensitivity [102]. Therefore, a promising direction is represented by therapeutic approaches based on EVs loaded with small interfering RNAs (siRNAs) targeting GB cells and downregulating the expression of the inherent oncogenic lncRNAs or miRNAs, thus inhibiting their transfer via EVs to other tumor and non-tumor cells within the GB microenvironment.

### 3.2. EVs as Drug Delivery Systems in GB Therapy

Recently, nanomedicine has dominated the field of tumor therapy with respect to different approaches, thanks to the abilities of nanoparticles to be loaded with specific therapeutic agents as well as surfaces decorated with specific targeting molecules. First, synthetic nanocarriers such as liposomes, have been used, due to their high loading and surface functionalization efficiency; however, they have important limitations, such as low tolerability, relatively short circulation times in biological environments, fast body clearance, and, last but not least, a low capability for BBB. Even though different strategies have been developed to increase BBB permeability and enhance nanoparticle access to the brain tissue, low biocompatibility and a high risk of off-target effects are still key issues to overcome [39,103].

EVs offer key advantages in this regard. Several studies have shown their low immunogenicity and cytotoxicity, low clearance from the phagocytic system, prolonged circulation time, high biocompatibility, and efficient cell uptake [104,105]. Interestingly, EVs are able to cross interspecies and even interkingdom boundaries; EVs isolated from different sources, including microalgae, bacteria, and plants, are easily uptaken by mammalian cells [106,107,108,109,110]. Through several physical/chemical/genetic strategies, EVs can be loaded with various functional cargoes, such as nucleic acids, proteins, natural substances, chemotherapeutics, photosensitizers, and sonosensitizers, that can be delivered to the cells. Moreover, the EV surface can be also functionalized with cell-type-specific targeting ligands to enhance the interaction with specific cellular types [39,111]. The encapsulation of therapeutic molecules within EVs can improve the molecules’ solubility, stability, bioavailability, and therapeutic effect. Furthermore, EVs are capable of crossing the BBB, making them the ideal candidates for delivering therapeutic molecules to the brain for the treatment of brain pathologies [103].

The first study on the ability of engineered EVs to cross the BBB and efficiently delivery functional molecules to the brain was performed by Alvarez-Erviti and coauthors by using EVs isolated from mouse bone-marrow-derived dendritic cells [112]. To confer brain-targeting capabilities, dendritic cells were previously engineered to express recombinant fusion proteins. These proteins contained the central nervous system-specific rabies viral glycoprotein (RVG) peptide, which specifically binds to the acetylcholine receptor, fused to the N terminus of a murine dendritic cell (DC)-derived lysosome-associated membrane protein (Lamp2b), which is a highly EV-associated protein. Isolated EVs exposing the RVG-Lamp2b fusion protein on the membrane were electroporated with specific siRNAs and then injected intravenously into mice. The EVs were capable of specific targeting and siRNA delivery to brain neurons, microglia, and oligodendrocytes [112]. A similar strategy was used in mouse models for Parkinson’s disease [113]. This approach has shown great potential in penetrating the BBB and could be exploited for the treatment of brain pathologies. Recently, Kim and coworkers [114] used the same Lamp2b-based approach to decorate EVs released from 293T cells with the Transferrin Receptor-binding peptide T7, which is able to target transferrin receptors naturally located on GB cells’ surface. The engineered T7-EVs were further modified to encapsulate antisense miRNA oligonucleotides against miR-21 (AMO-21). When injected intravenously into an intracranial GB xenotransplanted rat model, T7-EVs were much more efficient than both unmodified and RVG-decorated EVs in reaching the brain, targeting GB cells, and delivering AMO21, resulting in a reduction in GB size [114].

EVs from other cell types have also been successfully tested for GB treatment. EVs derived from a mouse lymphoma cell line (EL-4) were loaded with the anti-inflammatory drug curcumin or with an inhibitor of the signal transducer and activator of transcription 3 (STAT3). These EVs were efficiently and noninvasively delivered, via the intranasal route, to microglia cells in mice brains, causing a significantly delayed brain tumor growth in a glioma mouse model [115]. In addition, EVs from a brain endothelial cell line (b.END3), which are naturally enriched with the CD3 tetraspanin, were loaded with doxorubicin. These EVs were capable of crossing the BBB into a xenotransplanted zebrafish brain tumor model (obtained by injecting GB cells into the zebrafish brain ventricle), significantly decreasing the fluorescent intensity of xenotransplanted cancer cells and tumor growth markers [116]. 

MSCs are a well-studied source of EVs that have been utilized in several clinical trials, both in their native form or after engineering, to target a wide range of pathologies, including tumors [64,117]. MSCs can communicate with different cell types, including GB cells, through direct contact via gap junctions and/or contact-independent pathways [102,118]. Stromal cells resembling MSCs are a key component of the GB microenvironment, where they can play both tumor-supportive and -suppressive roles [119,120]. MSC-derived EVs carrying specific miRNAs (miR-7, miR-34a, miR-124, miR-133b, miR-145, miR-146b miR-199a, miR-375, or miR-584-5p) were found in vitro to counteract GB tumor features, including cell proliferation, migration, and invasion. Specific molecules or pathways were targeted by miRNAs, including Forkhead box (FOX)A2 (miR-124a), Wnt pathway (miR-133b), EGFR (miR-146b), SLC31A (miR-375), and MMP2 (miR-584-5p). Many of these MSC-EVs engineered with miRNAs were also tested in vivo in xenotransplanted mouse models, where they showed the ability to cross the BBB and promote tumor regression [100]. A very promising study is the one involving MSC-EVs loaded with miR-124a and systemically delivered in mouse models xenotransplanted intracranially with GB cells; the loaded MSC-EVs were able to suppress tumor growth and prolong overall animal survival [121].

Tumor-derived EVs (TDEVs) can be also used as natural carriers for the delivery of antioncogenic molecules [64], thanks to their high tumor targeting and permeability, making them a technological Trojan horse, as evoked by Simionescu and coauthors [100]. EVs isolated from engineered patient-derived GSCs and carrying the miR-302-367 cluster are rapidly internalized by neighboring GB cells, and are able to inhibit GB cell proliferation, stemness, and invasion through the repression of the Cyclin A, Cyclin D1, E2F1, and CXCR4 pathways [122]. In addition, GDEVs that were engineered with miR-124, miR-128, or miR-137 improved the survival rate of a GB mouse model when combined with chemotherapy [123], whereas GDEVs carrying miR-151a reduced chemoresistance in GB xenograft mouse models [124]. Cell treatment with miRNA-specific inductors can also be used as a valid alternative to external loading to obtain EVs carrying specific miRNA molecules. This is the approach used by Wang and coauthors, which employed traditional medicine substances to induce GDEVs’ miR-7-5p enrichment [125]. As a result, GDEVs carrying miR-7-5p reduced GB formation and metastasis in GB nude mouse models [125]. The exploitation of GDEVs as drug carriers for GB therapy requires an extremely careful evaluation of the antioncogenic properties of the loaded therapeutic molecule vs. the oncogenic potential of the GDEVs’ intrinsic cargo as well as a relative cost–benefit assessment. Guo and coworkers recently identified a saponin-mediated cargo elimination strategy to improve the biosafety of GDEVs in GB therapy applications [126]. A systematic analysis of the original proteins and RNAs together with functional in vitro and in vivo assays confirmed the high efficiency of the method in eliminating GB-EV cargo, and its inherited abilities in promoting GB progression, without affecting uptake by GB cells. Furthermore, saponin-treated GDEVs loaded with doxorubycin displayed an effective tumor-suppressive role in both subcutaneous and orthotopic GB mouse models [126]. These data are extremely promising and could be exploited to develop novel visionary therapeutic pipelines. These could involve isolating TDEVs directly from the patients, disarming their oncogenic properties through cargo elimination protocols, loading and/or functionalizing them with anticancer molecules, and administrating them to the patient. Finally, although TDEVs preferentially target (and are uptaken by) the cells of the same tumor type, TDEVs’ surface can be functionalized with specific molecules involved in EV–cell interactions to improve or alter natural tumor tropisms. Geng and coauthors have shown that GDEVs are more easily uptaken by GB cells than other type of tumor cells such as pancreatic cancer (PC) cells, and vice versa [127]. However, functionalizing EVs with cyclic arginine-glycine-aspartic acid-tyrosine-cysteine (cRGDyC), a ligand for the integrin αvβ3 enriched in GB cells, enhances the ability of PC cells (and also the GB cells themselves) to target and be internalized into GB cells [127]. 

Other cells that naturally show GB tropism are neural stem cells (NSCs), from which the GB might be derived [57,58], and teratocarcinoma cells. Both NSCs and NTERA2 human teratocarcinoma cells, which resemble NSCs as they can differentiate into both glia and neurons [128,129], demonstrate GB tropism in vivo and have been proposed for cell-based delivery systems in anti-GB therapies [130,131,132]. EVs released by NSCs and engineered with antisense oligonucleotides (ASOs) targeting oncogenic and tolerogenic signal transducers and activators of transcription 3 (STAT3) were effective, after intracranial injection at a distant site, in reaching the glioma microenvironment, targeting and activating microglia to inhibit tumor growth [133]. Even more interestingly, native NTERA2-derived EVs, naturally carrying the oncodevelopmental factor Cripto [134,135,136], have been recently shown to impair GB cell migration in vitro without inducing undesirable effects such as increased GB cell proliferation or enhanced TMZ drug resistance [137]. Cripto is a membrane-bound glycosylphosphatidyl inositol-anchored protein that can be also cleaved and released as a soluble factor [138], and it only recently has been found to be associated with EVs [137,139]. Mouse *Cripto* gene targeting was associated with neural differentiation in embryonic stem cell cultures, the specification of anterior neural identities in vivo, and *Cripto* null embryos, which mostly comprise anterior neuroectoderm, including NSCs [140,141,142]. Cripto has been mostly identified as being associated with oncogenic features, particularly in GB, but it is also able to act as an antioncogene [143,144,145,146]. Although these data have not yet been confirmed in vivo, they suggest new unobvious and even paradoxical paradigms in GB, as well as in tumor in general and their progression and therapies. First, EV sorting and delivery could be an alternative route for regulating the spread and activity of soluble and/or membrane-bound signaling molecules, modulating their final impact on target cells and then on cancer development and progression [64,137]. Secondly, specific subsets of TDEVs might possess antitumoral potential and could be used per se or eventually enhanced through ad hoc modifications [64]. Therefore, molecularly identifying and isolating these specific subsets of TDEVs, and then inducing or bio-mimicking them is certainly a novel, ambitious, and promising therapeutic direction to explore. 

## 4. Mitigating the Pitfalls and Realizing the Potential of EV Exploitation in GB Treatment

**Figure 3:**
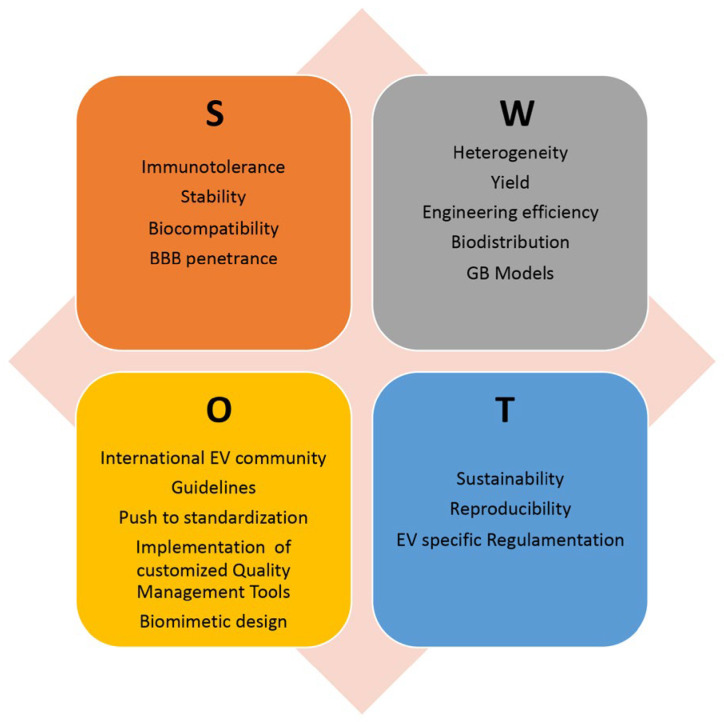
SWOT analysis of EV exploitation in GB treatment, highlighting the relative Strengths (S), Weaknesses (W), Opportunities (O), and Threats (T). BBB: blood-brain barrier; GB: glioblastoma.

Despite these promising results, there are currently no ongoing clinical trials for EV-based GB treatment; however, EV-based therapies are being evaluated in clinical trials for the treatment of other types of tumors. This paragraph outlines the main issues and directions to explore in order to find the optimal solutions and fully realize the potential of EVs as an advanced therapeutic platform for GB treatment. Figure 3 shows an analysis of the Strengths, Weaknesses, Opportunities, and Threats (SWOT) of using EVs in novel GB therapeutic strategies. 

### 4.1. Extracellular Vesicle Source

The previous subsection has listed the main EV sources that have been used for GB therapeutic assays both in vitro and in vivo. They are all derived from mammalian cells, including cell lines of brain endothelial cells, tumor cells (both GB and teratocarcinoma), and stem cells (both neural and mesenchymal). To be exploited in patient therapy, EVs must be produced in high quantities and with extremely high quality, thereby complying with good manufacturing practices (GMP), pharmaceutical biologics regulation, and ongoing specific regulations for EV therapeutics [106]. The process needs careful scaling up and out, standardization, and quality assurance of the experimental pipeline, as well as quality control of the final products, requiring a high commitment in terms of cost, resources, and time. Alternative sources of EVs, such as plants, microalgae, and bacteria, have also been identified, and their biocompatibility and biodistribution in animal models have been studied [106]. These alternative EV sources have a lower cost of production, but unfortunately, no brain tropism has been proved yet. Therefore, the production pipeline must be extended to include an EV surface functionalization phase, followed by the isolation and characterization of the engineered EVs. A careful evaluation of the advantage and disadvantages of each strategy must be performed, in terms of therapeutic efficacy and efficiency as well as of cost and sustainability.

### 4.2. Extracellular Vesicle Handling Procedures

Various techniques have been used until now for EV isolation, including differential ultracentrifugation (dUC), which is still considered the gold standard, as well as different commercial kits, exclusion chromatography, and tangential flow filtration [147]. EV loading is usually subdivided into endogenous or indirect (the manipulation of the parental cell) and exogenous or direct (EV engineering), the latter of which can be further subdivided into passive (co-incubation of molecules and EVs) and active (i.e., electroporation and sonication) [148]. Standardized procedures should be identified to ensure low inter-batch variability and the high reproducibility of the results. The International Society for EVs (ISEV) has been committed to this direction for years, providing the EV scientific community with minimal information for studying EVs and several position papers dedicated to specific aspects of EV research and exploitation [149,150,151,152,153]. Recently, it is becoming increasingly evident how quality management tools, which have already been used in other scientific contexts [154,155,156], can assist in resolving the main issues in EV research and development [157,158]. Applying the Failure Mode and Effect Analysis, for the risk assessment and management of scientific procedures [159] to the individual steps of the EV production pipeline might help in avoiding and preventing pitfalls and failures in EV bioprocessing and manipulation [158,160]. Similarly, the Design of Experiments mathematical model [161,162] might be used to identify and optimize key factors in multivariable processes, such as production, engineering or functional assays of EVs [158,160,163]. Recently, a new tool, called EV decision-making grid (EV-DMG), inspired to multicriteria decision making, has been proposed as a novel, customizable, efficient and easy-to-use model to support responsible EV research and innovation [164]. All together, these instruments and guidelines can greatly improve the selection, optimization, standardization, quality assurance and control, and ultimately the reliability of the EV landscape.

### 4.3. EV Biodistribution 

The biodistribution of EVs was evaluated and found to be concentrated in the spleen, liver, lungs, kidneys, and gastrointestinal tract [165]. TDEVs, instead, have a high tropism towards tumor cells, especially those of the same type. Additionally, the final biodistribution of EVs can be influenced by the source and handling of EVs as well as the “ad hoc” cell surface functionalization [166]. EV size is also an important variable to consider: smaller EVs are easily eliminated via blood circulation, whereas larger EVs may have difficulties in passing through the BBB but are more efficient in delivering their cargo content [39,102]. EVs have been shown to penetrate the BBB and deliver therapeutic agents into the brain, making them suitable drug delivery vectors for intravenous administration. Determining the appropriate quantity of EVs to administer and the frequency of administration requires an evaluation of the amount of EVs that can pass through the barrier and enter to the brain, as well as the amount of EV cargo that can be effectively delivered to the GB cells. However, as GB therapy typically involves surgical tumor resection as a first step, it may also be a suitable strategy to concomitantly inject therapeutic EVs at the tumor site to prevent recurrence. 

### 4.4. Biomimetic Nanoparticles

Recently, an important research direction is exploring the possibility of obtaining biomimetic nanocarriers that combine the advantages of both artificial (e.g., high yield and loading efficiency) and natural (e.g., low immunogenicity, biosafety, and biocompatibility) delivery vectors through the development of technologically advanced ad hoc nanoplatforms. Several methods can be used to create EV-like biomimetic nanoparticles (EBPs), including parental cell loading and consequent extrusion, which is the most common. This also includes the possibility of wrapping organic or inorganic synthesized nanoparticles in a cell membrane, by which the biocompatibility and targeting properties of the parental cells are transferred to the obtained EBPs [167,168,169]. Biomimetic nanovesicles were obtained by subjecting brain endothelial cells to serial extrusion through filters with diminishing pore sizes, after the cells had been loaded with chemotherapeutic agents. The resulting EBPs had a similar drug-loading capacity and pharmacokinetics profiles to EVs naturally produced by the same cells and were able to cross the BBB in orthotopic xenograft mouse models, but they achieved a higher yield than natural EVs [170,171,172]. Recently, a nanoplatform called EV-DNs was developed, in which doxorubicin-loaded heparin nanoparticles (DNs) were attached to the surface of native grapefruit EVs. EV-DNs were capable of penetrating the BBB in vivo, reduce GB cell proliferation and increase animal model survival [108]. Another innovative biomimetic design developed for targeting GB included the co-loading of the antitumoral Cu synthetic chelator di-2-pyridylketone-4,4-dimethyl-3-thiosemicarbazone (Dp44mT) with Regadenoson (Reg), which transiently opens the BBB, and lastly, Angiopeptide-2 functionalized red blood cell membrane (Ang-M) camouflaging. When injected into orthotopic xenografted mouse models, the obtained Ang-M@(Dp44mT/Reg) nanoparticles actively traversed the BBB and delivered Dp44mT specifically to GBM cells with negligible systemic drug toxicity [173]. 

Finally, EBPs are a promising delivery platform for cancer combination therapy, which has emerged as a relevant trend in cancer and GB treatment. Biomimetic nanovesicles can be derived from GB cells and used for the encapsulation of both photosensitizers (i.e., berberine) and chemotherapeutics (i.e., Temozolomide and Givinostat) to combine different drugs and approaches, thus achieving higher therapeutic effects [174]. Biomimetic theranostic nanovectors composed of iron-modified polydopamine nanoparticles loaded with the anticancer drug doxorubicin (MPDAFe@DOX) and coated with cancer cell membranes (MPDAFe@DOX@Mem) were found to be effective in killing cancer cells through a combination of chemotherapy and PDT while also enabling MRI imaging [175]. A biomimetic system, named RGE-Exo-SPION/Cur, was implemented by loading curcumin (Cur) and superparamagnetic iron oxide nanoparticles (SPIONs) into EVs further functionalized by click chemistry to be carried on the surface of a neuropilin-1-targeted peptide (RGERPPR, RGE). The RGE-Exo-SPION/Cur nanoparticles proved effective in targeting GB cells and reducing GB tumor size in orthotopic GB-bearing nude mouse models, and they were also relevant for simultaneous GB MRI-based diagnosis and therapy [176]. Combination cancer therapy utilizing biomimetic nanoparticles and sonodynamic-enhanced chemotherapy has demonstrated synergistic antitumor efficacy and significantly prolonged the survival rate of mouse models. The biomimetic nanosonosensitizer systems were fabricated with biodegradable and pH-sensitive polyglutamic acid (PGA) and the chemotherapeutic agent and sonosensitizer doxorubicin (DOX), camouflaged with human GB U87 cell membranes. The resulting EBPs in combination with noninvasive ultrasounds were capable of passing through the BBB, induce cancer cell apoptosis, and increase chemosensitivity in orthotopic GBM xenograft mouse models [177].

### 4.5. Glioblastoma Models

One of the main reasons for the limited success of novel therapeutic molecules entering clinical trials is the poor correlation between their efficacy in in vitro, ex vivo, and in vivo tumor models, and the patient treatment scenarios. Several in vitro and ex vivo models have been developed to study GB, each one with its own strengths and weaknesses, ranging from conventional GB cell lines to emerging technologies, such as bioprinting and microfluidics [7,178]. In 2006, primary early passage cells from fresh GB biopsies were isolated. The maintenance of cells in serum-free supplemented medium preserved the genetic heterogeneity and gene expression profiles of the tumor of origin [179]. GB-patient-derived cell lines (GPDCLs) were cultured in both bi-dimensional (2D) and three-dimensional (3D) cell cultures (spheroids or gliomaspheres) as well as used for mouse xenografts [7,178]. In 2015, a biobank of 48 GPDCLs representing all four transcriptional GB subtypes was created and made freely available [180]. Recently, a reference collection of 12 GPDCLs, the different molecular subtypes, and relative xenograft mouse models was developed for the identification and evaluation of novel GB therapeutics [181]; however, establishing and maintaining these cells in vitro as well as growing them in vivo in mouse models is challenging and limits their utility [182]. Furthermore, 3D structures that include not only GB cells but also non-tumor cells can mimic the GB tumor microenvironment and are definitely better models for therapeutic studies. In this context, organotypic glioma slice cultures derived directly from tumor biopsies have the great advantage of comprising several cell types and structures of the GB tumoral microenvironment. They were used to study GB cell proliferation, migration, invasion, and susceptibility to treatment [7,178,183]. In 2016, the first GB organoids were obtained by adapting the methodology used to develop cerebral organoids or mini-brains from induced pluripotent stem cells to GB cells [184,185]. These organoids were successfully implanted into mouse brains, resulting in models that were more sophisticated and representative of the parental tumors than simple GPDCLs [185]. A biobank of patient-derived glioblastoma organoids, with each one showing parental tumor heterogeneity, was establish and is of high value for identifying personalized target and treatment strategies [186]. However, GB organoids self-assemble, resulting in high variability in cellular composition and structure. Recently more and more sophisticated and high-cost models have been developed based on emerging technologies such as bioprinting and microfluidics. 3D bio-printed GB models exploit novel biomaterials and tissue engineering methodologies to reconstruct 3D models of the tumors on the basis of clinical images. This allows for a high control over both cellular and the extracellular matrix disposition and the high level of homogeneity between the bio-printed models [187,188]. Microfluidic devices take advantages of a central chamber where tumor cells are cultured in a 3D collagen hydrogel perfused with microliters of circulating media whose composition is controlled in terms of nutrients, pH, and growth factors to mimic vasculature and create a more realistic and dynamic GB microenvironment [189,190,191] Microfluidics has also been applied to enrich and characterize specific GB cells and also GDEVs [192,193]. This technology has many advantages, such as simplified on-chip processing, guaranteeing minimal cell samples, high sensitivity, and rapid analysis [178]. Furthermore, microfluidics, tissue engineering, and biomaterials research have been converging to develop more sophisticated 3D cancer-on-a-chip tissue models with the potential to significantly advance the understanding of cancer biology and allowing for accelerated and cost-effective drug discovery [194].

Finally, to assess the ability of therapeutic EVs to efficiently deliver therapeutic molecules across the BBB, without inducing immune reactions or other undesired systemic effects, in vivo studies are absolutely needed. Orthotopic xenotransplanted immunocompetent mouse models have been shown to recapitulate patient GB–microenvironment interactions and are therefore suitable for this purpose [195,196]. Recently, zebrafish xenografts have been proposed as an alternative in vivo model due to several advantages. The transparency in the early stages, which allows easy the visualization of the tumor; the high number and rapid development of offspring; and the small size make them extremely suitable for high-throughput screening [197,198,199].

In conclusion, there is no single optimal model that is suitable for all needs, but researchers have to carefully select the best model(s) for their specific study purpose. Ad hoc grants, collaborations, and access to platforms for testing specific GB models will help in the collection of more and more robust data and strengthen preclinical studies in a short time. 

## 5. Conclusions

GB is a rare but extremely aggressive brain tumor that has a significant impact on patient outcomes, affecting both the duration and quality of life, and no breakthrough therapies have yet been discovered that can prolong patient survival. Functional and preclinical studies based on the isolation and engineering of EVs from different cellular sources, as well as the design and production of more complex and sophisticated EBPs, are definitely a promising direction to explore. Furthermore, EVs and EBPs could efficiently deliver more than one anticancer molecule with different mechanisms of action to GB cells, and other cells within the GB microenvironment, to synergistically enhance their therapeutic effects. The use of EVs and EBPs, in combination with chemotherapy, photodynamic, as well as sonodynamic therapies, might have a desirable higher impact on GB patients’ survival, relapse-free time, and quality of life. In addition, the combination of different ex vivo and in vivo models of GB may contribute to the identification and study of the most promising anti-GB drugs and the production of more robust and reliable preclinical data. This could help to increase the success rate of subsequent clinical trials and advance EV-based anti-GB combination approaches into the GB therapeutic pipeline.
